# Do preserved foods increase prostate cancer risk?

**DOI:** 10.1038/sj.bjc.6601755

**Published:** 2004-04-27

**Authors:** L Jian, D H Zhang, A H Lee, C W Binns

**Affiliations:** 1 School of Public Health, Curtin University of Technology, GPO Box U1987, Perth, WA 6845, Australia; 2 Zhejiang University School of Medicine, 310006 Hangzhou, China

**Keywords:** preserved food, prostate cancer, case–control study, China

## Abstract

Preserved foods have been found in some studies to be associated with increased cancer risks. The possible relationship between preserved foods and prostate cancer was investigated in a case–control study in southeast China during 2001–2002 covering 130 histologically confirmed cases and 274 inpatient controls without malignant disease. The total amount of preserved food consumed was positively associated with cancer risk, the adjusted odds ratio being 7.05 (95% CI: 3.12–15.90) for the highest relative to the lowest quartile of intake. In particular, the consumption of pickled vegetables, fermented soy products, salted fish and preserved meats was associated with a significant increase in prostate cancer risk, all with a significant dose–response relationship.

The incidence of prostate cancer exceeds that of all other cancers among men in North America, Australia, New Zealand and most Northwestern European countries, whereas it is low in Asian countries (IARC, 2001). The large differences in incidence between countries, together with comparative research on Asia immigrants, suggest that lifestyle and environmental factors may contribute to the etiology of prostate cancer (Tominaga and Kuroishi, 1997).

A major environmental factor is diet though, despite many studies, the effects of dietary factors and food processing methods on prostate cancer remain uncertain. In a cohort study in the Netherlands, in which 642 cancer cases occurred by the end of follow-up consumption of cured meat was associated with an increased prostate cancer risk after adjusting for age, socioeconomic status and family history (Schuurman *et al*, 1999). However, another case–control study conducted in Poland using 76 cases and 152 controls reported that smoked fish consumption was associated with half the risk of those who seldom ate this (Pawlega *et al*, 1996).

To assess the relationship between preserved foods and the risk of prostate cancer, a case–control study was conducted in Zhejiang Province located in southeast China, where the incidence rate of prostate cancer is still low at 1.7 per 100 000 (IARC, 2001).

## MATERIALS AND METHODS

### Study design and participants

A hospital based 1 : 2 case–control design was used. Cases were identified by daily searches of all inpatient records and pathology reports in the urology wards of the eight public hospitals in Hangzhou (capital of Zhejiang Province), between July 2001 and June 2002. Inclusion criteria for cases were defined to be men over 45 years of age with a confirmed histopathological report of adenocarcinoma of the prostate, who had been residents of Zhejiang Province for at least 10 years and were capable of being interviewed. Potential cases with a diagnosis of Alzheimer's disease or a history of stroke were excluded to avoid recall bias. Of the 143 cases identified during the period, 133 (93%) were interviewed and 10 (7%) declined to participate in the study. Three patients were later excluded because their date of initial diagnosis was more than 3 years ago. Most of the final 130 cases (84%) were recent patients interviewed within 12 months from diagnosis. The distribution of cases by their stage was: A (12), B (28), C (37), D (52) and missing (1).

During the same period, 274 inpatient controls were recruited from the same eight hospitals and interviewed; they came from the same catchment area as the cases, were matched by age (±5 years), had no malignant disease confirmed by physical examination, X-ray, operation or histopathological reports. The exclusion criteria on Alzheimer's disease and stroke also applied to controls. The controls were recruited from the urology (65.3%), orthopaedic and trauma (19.3%), and colon and rectum surgery wards (15.4%) after confirmation of their diagnoses. Of the 284 eligible controls identified, 274 (96.5%) participated in the study, eight (2.8%) declined the interview and two persons (0.7%) later withdrew for personal reasons.

### Questionnaire

A structured questionnaire was used to collect information on demographic characteristics, height and weight, physical activity, usual diet, medical history and family history of prostate cancer, and factors related to marital status and reproductive factors. A reference recall period was set at 5 years before diagnosis for cases or 5 years before interview for controls. The quantitative Food Frequency Questionnaire (FFQ) component on habitual diet was modified from that used in the Shanghai stomach cancer study (Ji *et al*, 1998) and our previous ovarian cancer study (Zhang *et al*, 2002), which in turn had included components from the Hawaii Cancer Research Survey (Goodman *et al*, 1997), the Australian Health Survey 1995 (Australian Bureau of Statistics, 1995) and the USA food survey (National Information Services (NIS), 1992). These questionnaires have been validated in studies of large multiethnic populations including Chinese immigrants and native Chinese who were comparable to our study population of Zhejiang Chinese men (Goodman *et al*, 1997; Zhang *et al*, 2002).

The FFQ contained questions on 130 food items, which included all the foods in the usual diet of Zhejiang residents. Information was sought on the quantities of each food consumed per meal, including preserved food items, which are listed in the appendix. Food intakes were categorised as 0–2 times a year, 3–11 times a year, once a month, 2–3 times a month, once a week, 2–3 times a week, 4–6 times a week, once a week, once a day and ⩾2 times a day.

### Ethics and interview

The study was approved by the Human Research Ethics Committee of Curtin University of Technology and the Zhejiang hospital administration, as well as the doctors-in-charge of the relevant wards. Confidentiality and anonymity issues were explained to each participant and formal consent was sought prior to the face-to-face interview. The first author conducted all interviews, usually in the presence of the participant's next-of-kin to minimise recall bias. Each interview usually took an hour to complete. Histopathological records were obtained from the pathology department or retrieved from inpatient medical records of the relevant eight hospitals.

### Statistical analysis

All data were coded and analysed using the SPSS package. The frequency and quantity variables were expressed in terms of quantities of foods consumed per day (g day^−1^). Adjustments were made for the edible portions of foods (e.g. rice 100%, pea 42%, apple 76%), seasonal factors and market availability (Whittemore *et al*, 1990). The latter figures represented the average availability period of the food supply over a normal year (e.g. radish 5 months, pea 2 months, celery 9 months). The total energy intake from the 130 food items was calculated based on the *Table of Food Components* (Institute of Nutrition and Food Hygiene, Chinese Academy of Preventive Medicine, 2000).

To assess potential survival bias, data for the 109 recent patients (interviewed within 1 year from diagnosis) and data for all cases (interviewed within 3 years from diagnosis) were analysed separately. Demographic characteristics and potential risk factors between cases and controls were compared by *t* test for continuous variables and χ^2^ test for categorical variables. To facilitate statistical analysis, each preserved food variable was categorised into three or four levels according to the distribution among the controls, with the lowest level of intake taken as the reference category.

Crude and adjusted odds ratios (OR) and associated 95% confidence intervals (CI) of risk for preserved food variables were obtained from fitting unconditional multivariate logistic regression models. Each fitted equation included terms for adjusting age at interview, body mass index (BMI), overall physical activity level, locality of residence, education, family income, marital status, family history of prostate cancer, total caloric intake (kcal day^−1^), tea drinking, fresh vegetables and fruits intake. These variables were included because they were either plausible risk factors from the literature (Stewart and Kleihues, 2003) and our previous study (Jian *et al*, 2004) or potential confounders according to the univariate analysis. Overall physical activity was measured in terms of metabolic equivalent tasks (MET), with scores 1.5, 3.0 and 6.0 assigned for sedentary, moderate and vigorous activities, respectively to generate weighted estimates of time spent each week in physical activities (Ainsworth *et al*, 2000; Lacey *et al*, 2001).

## RESULTS

Table 1

**Table 1:** Characteristics of prostate cancer cases and controls

	**Cases (*n*=130)**	**Controls (*n*=274)**
Age at interview, mean years (s.d.)	72.7 (7.1)	71.4 (7.2)
BMI*, mean kg m^−2^ (s.d.)	23.4 (3.1)	22.7 (3.1)
MET, mean (s.d.)	157.4 (59.2)	156.5 (55.4)
Caloric intake, mean kcal day^−1^ (s.d.)	2386.4 (809.3)	2332.9 (619.4)
Fresh vegetables and fruits*, mean g day^−1^ (s.d.)	499.7 (320.8)	865.2 (477.6)
Fresh meat and fish, mean g day^−1^ (s.d.)	81.7 (54.8)	85.0 (54.1)
		
*Locality of residence, n* (%)		
Urban	97 (74.6)	207 (75.5)
Rural	33 (25.4)	67 (24.5)
		
*Education, n* (%)		
No formal education	18 (13.8)	29 (10.6)
Primary	37 (28.5)	78 (28.5)
Secondary	44 (33.8)	110 (40.1)
Tertiary	31 (23.8)	57 (20.8)
		
*Income per month, RMB* (%)		
⩽500	24 (18.5)	50 (18.2)
501–1000	55 (42.3)	113 (41.2)
1001–2000	43 (33.1)	99 (36.1)
>2000	8 (6.2)	12 (4.4)
		
*Marital status, n* (%)		
Married	114 (87.7)	245 (89.4)
Widowed, divorced, separated	16 (12.3)	29 (10.6)
		
Prostate cancer in first degree relatives*, n (%)		
No	111 (85.4)	241 (88.0)
Yes	3 (2.3)	0 (0.0)
Unclear	16 (12.3)	33 (12.0)
		
*Alcohol consumption, n* (%)		
Never	60 (46.2)	142 (51.8)
Moderate	38 (29.2)	70 (25.5)
Heavy a	32 (24.6)	62 (22.6)
		
*Tobacco smoking, n* (%)		
Never	44 (33.8)	103 (37.6)
Former	66 (50.8)	107 (39.1)
Current	20 (15.4)	64 (23.4)
		
Drink tea*, n (%)		
No	58 (44.6)	55 (20.1)
Yes	72 (55.4)	219 (79.9)

* *P*<0.05.

a Score of drinks/week >25 classified as heavy drinkers.

contrasts the sample characteristics of men with and without prostate cancer. There were no significant differences between cases and controls in mean age at interview, overall physical activity (MET), locality of residence (urban or rural areas), education, family income, marital status, alcohol consumption and tobacco smoking. The two groups were also similar in terms of total caloric intake, and fresh meat and fish consumption. However, the cases had a higher BMI and more often family history of prostate cancer; they also drank less tea and ate less fresh vegetables and fruits than the controls.

Table 2

**Table 2:** Preserved foods and prostate cancer risk

	**No. of cases (%)**	**No. of controls (%)**	**Crude OR**	**95% CI**	**Adjusted OR^a^**	**95% CI**
*Total preserved food* (*g day*^−1^)						
⩽6.85	12 (9.2)	69 (25.2)	1.00		1.00	
6.86–13.86	13 (10.0)	68 (24.8)	1.10	0.47–2.58	1.08	0.41–2.86
13.87–26.24	28 (21.5)	69 (25.5)	2.33	1.10–4.96	2.32	0.96–5.65
>26.24	77 (59.2)	68 (24.8)	6.51	3.25–13.03	7.05	3.12–15.90
*P* for trend						0.000
						
*Pickled vegetables* (*g day*^−*1*^)						
<1.56	12 (9.2)	68 (24.8)	1.00		1.00	
1.56–4.11	12 (9.2)	75 (27.4)	0.91	0.38–2.15	0.77	0.28–2.09
4.12–9.26	16 (12.3)	63 (23.0)	1.44	0.63–3.28	1.77	0.66–4.74
>9.26	90 (69.2)	68 (24.8)	7.50	3.76–14.94	10.06	4.23–23.94
*P* for trend						0.000
						
*Fermented soy products* (*g day*^−*1*^)						
0	51 (39.2)	126 (46.0)	1.00		1.00	
0.10–4.00	30 (23.1)	96 (35.1)	0.77	0.46–1.30	0.77	0.41–1.42
>4.00	49 (37.7)	52 (19.0)	2.33	1.40–3.87	2.02	1.08–3.78
*P* for trend						0.003
						
*Salted fish* (*g day*^−*1*^)						
0	59 (45.4)	152 (55.5)	1.00		1.00	
0.10–0.88	24 (18.5)	58 (21.2)	1.07	0.61–1.87	1.00	0.51–1.93
>0.88	47 (36.2)	64 (23.4)	1.89	1.17–3.06	2.12	1.17–3.86
*P* for trend						0.014
						
*Preserved meats* (*g day*^−*1*^)						
<0.44	19 (14.6)	68 (24.8)	1.00		1.00	
0.44–1.37	33 (25.4)	98 (35.8)	1.21	0.63–2.29	0.82	0.38–1.76
1.38–3.42	26 (20.0)	40 (14.6)	2.33	1.15–4.73	2.32	1.00–5.38
>3.42	52 (40.0)	68 (24.8)	2.74	1.47–5.11	2.46	1.17–5.17
*P* for trend						0.010

a Estimates from multivariate logistic regression models included terms for age at interview, BMI (kg m^−2^), physical activity (MET), locality of residence (urban, rural), education (none, primary, secondary, tertiary), family income per month (RMB; ⩽500, 501–1000, 1001–2000, >2000), marital status (married, widowed or divorced or separated), prostate cancer in first-degree relatives (no, yes, unclear), caloric intake (kcal day^−1^), fresh vegetables and fruits consumption (g day^−1^), tea drinking (yes, no).

presents the results from separate multivariate logistic regression fits for each preserved food consumption measure. The total amount of preserved food consumed was positively associated with cancer risk, the adjusted odds ratio being 7.05 (95% CI: 3.12–15.90) for the highest quartile relative to the lowest quartile of intake. The cancer risk tended to increase with increasing consumption of pickled vegetables, fermented soy products, salted fish and preserved meats, all with significant dose–response relationships. In particular, the effect of pickled vegetables was substantial among the preserved food subgroups. Results from the 109 recent patients were also similar (data not shown).

## DISCUSSION

This is the first report of a link between preserved foods and the risk of prostate adenocarcinoma. Much effort was made to obtain accurate information on dietary exposure.

For centuries foods have been preserved by salting and other processes such as pickling and curing that use salt. Salted foods are common in China, Korea and Japan, though the methods of salting vary (Potter *et al*, 1999). The traditional diet of Zhejiang senior citizens contains local pickled vegetables, and about 75% of the controls and 90% of the cases in our study consumed at least 1.6 g of pickled vegetables daily. Men consuming in excess of 9 g daily (70% in case group and 25% in control group) had a significantly high cancer risk even after accounting for their intake of fresh vegetables and fruits and other confounding factors.

The mechanism of deleterious effects of preserved foods remains unclear. However, a recent toxicological study in Hangzhou (Yuan and Ding, 2003) found among six traditional preserved foods, three items (salted mustard greens, salted fish and salted pork meat) showed significant mutagenic activity in cell micronucleus test (Yuan and Ding, 2003). It has been reported that diets high in cured meats possibly increase the risk of colorectal cancer (Potter *et al*, 1999), whereas salted foods may increase the risk of colon (Chiu *et al*, 2003), stomach (Ji *et al*, 1998), oesophageal (Gao *et al*, 1994) and ovarian cancer (Zhang *et al*, 2002). A case–control study in Japan found intakes of miso soup (fermented soybean paste) was associated with an elevated lung cancer risk (Wakai *et al*, 1999), a study in Japan has suggested a link between gastric cancer and the consumption of picked vegetables (Huang *et al*, 2000).

Several issues are relevant to our findings. Although a case–control design appears appropriate because of the relatively low incidence of prostate cancer in China, this is subject to a number of potential biases. Substantial effort was made to assess of exposures, by collecting extensive details of preserved food intake and dietary patterns. Our previous study using a similar recruitment strategy and reference recall period found no differences in food consumption pattern between hospital-based controls and community controls (Zhang *et al*, 2002), so that the participants recruited may be considered representative of the Zhejiang population. In spite of adjusting for confounding factors in the multivariate analysis, possible residual confounding by other (as yet unidentified) dietary habits cannot be ruled out. With regard to potential sources of biases, selection or recruitment bias appeared to be minimal in view of the low refusal rate among participants. The majority of cases were recently diagnosed, while the study design ensured that ascertainment of cases was complete. Survival bias was found to be minimal. The association between preserved food consumption and prostate cancer had not been established at the time of interview and participants were blinded to the purpose of the study. In addition, a reference recall period (5 years before diagnosis for cases and 5 years before interview for controls) was adopted to avoid possible change in food consumption patterns since the onset of the disease. To increase the accuracy of the FFQ, a series of standard containers were used to quantify the intake of each food item. Most of the interviews were conducted in the presence of participant's next-of-kin to assist in recall. Finally, a single investigator (the first author) conducted all interviews following exactly the same procedure for both cases and controls to avoid intra- and inter-interviewer biases.

In conclusion, the evidence from Chinese men suggests that consumption of preserved foods may increase the risk of prostate cancer.

## Appendix

See Table A1

**Table A1:** Composition of food groups by plant and animal sources

**Food group**	**Composition**
*Plant sources*	
Fresh vegetables	Greens, spinach, cabbage, Chinese cabbage, cauliflower, celery, bean sprouts, eggplant, wild rice stem, lettuce, pumpkin, white gourd, cucumber, carrot, fresh mushrooms, sweet green/red peppers, tomato, bamboo shoot, lotus root, dishcloth gourd, Chinese wax gourd, white radish, taro, sweet corn, garlic, garlic stalks, Chinese chives, leek, onion, spring onion, ginger, green/red fresh chilli, kelp, sea weeds, legumes and products soybean and products, green beans and peas, potato, sweet potato
Fresh fruits	Apple, pear, orange, tangerine, banana, grape, watermelon, peaches, pineapple, strawberries, plums, apricot, dates
Pickled vegetables	Fresh salted bok choy, salted mustard greens, salted bamboo stalk (dry), pickled radish, fermented amaranth peduncle, chowchow, gherkin
Fermented soy products	Fermented bean-curd, stinking bean-curd
Tea	Green tea, black tea
	
*Animal sources*	
Fresh meat and fish	Pork feet, pork (fat), pork (lean), pork (fat and lean), pork liver, other organ meats, beef and mutton, chicken, duck, sea fish (e.g. hairtail, yellow croakers), river fish (e.g. silver carp, crucians, herring, chub)
Preserved meats	Sausages, ham, bacon
Salted fish	Salted sea fish and river fish

for composition of food groups by plant and animal sources.
